# Improvement of the Nutraceutical Profile of Brewer’s Spent Grain after Treatment with *Trametes versicolor*

**DOI:** 10.3390/microorganisms10112295

**Published:** 2022-11-19

**Authors:** Anđela Zeko-Pivač, Anja Bošnjaković, Mirela Planinić, Jelena Parlov Vuković, Predrag Novak, Tomislav Jednačak, Marina Tišma

**Affiliations:** 1 Faculty of Food Technology Osijek, Josip Juraj Strossmayer University of Osijek, F. Kuhača 18, HR-31 000 Osijek, Croatia; 2 NMR Centre, Ruđer Bošković Institute, Biljenička 54, HR-10000 Zagreb, Croatia; 3 Department of Chemistry, Faculty of Science, University of Zagreb, Horvatovac 102a, HR-10000 Zagreb, Croatia

**Keywords:** brewer’s spent grain, solid-state fermentation, *Trametes versicolor*, enzyme cocktail, phenolic compounds

## Abstract

Brewer’s spent grain (BSG) is an important secondary raw material that provides a readily available natural source of nutraceuticals. It finds its largest application as animal feed and part of the human diet, while the future perspective predicts an application in the production of value-added products. In order to investigate a sustainable BSG treatment method, two BSG samples (BSG1 and BSG2) were evaluated as substrates for the production of hydrolytic (xylanase, β-glucosidase and cellulase) and lignolytic enzymes (laccase, manganese peroxidase and lignin peroxidase) by solid-state fermentation (SSF) with *Trametes versicolor* while improving BSG nutritional value. The biological treatment was successful for the production of all hydrolytic enzymes and laccase and manganese peroxidase, while it was unsuccessful for the production of lignin peroxidase. Because the two BSGs were chemically different, the *Trametes versicolor* enzymes were synthesized at different fermentation times and had different activities. Consequently, the chemical composition of the two BSG samples at the end of fermentation was also different. The biological treatment had a positive effect on the increase in protein content, ash content, polyphenolic compounds, and sugars in BSG1. In BSG2, there was a decrease in the content of reducing sugars. Cellulose, hemicellulose, and lignin were degraded in BSG1, whereas only cellulose was degraded in BSG2, and the content of hemicellulose and lignin increased. The fat content decreased in both samples. The safety-related correctness analysis showed that the biologically treated sample did not contain any harmful components and was therefore safe for use in nutritionally enriched animal feed.

## 1. Introduction

Brewer’s spent grain (BSG) is the main solid waste stream of the brewing industry. A total of 20 kg of BSG is produced per 1 hL of brewed beer. It is estimated that about 36.4 million tons of BSG are available in the world per year [1]. Chemically, BSG is a lignocellulosic material, mainly composed of hemicellulose, cellulose, and lignin, but also contains proteins, lipids, vitamins, minerals, and polyphenolic compounds [1,2,3]. BSG can be, therefore, considered as a potential raw material for various purposes, rather than a waste [4]. BSG is widely used as animal feed, although it has limited digestibility for animals due to the high content of dietary fiber. Improving the nutrient profile of BSG could lead to its larger integration into feed and human nutrition systems and provide several health benefits [5]. Moreover, with the help of appropriate processing methods, BSG can be used not only for the production of higher-quality feed and food, but also for bioenergy and fertilizers, as well as in waste management [1].

Nutraceuticals are compounds derived from natural sources that are becoming a growing trend in health and nutrition, replacing various drugs (non-steroidal anti-inflammatory drugs, analgesic [6], antihypertensive drugs [7], and dietary supplements). Among the wide variety of nutraceuticals such as carbohydrate derivatives, fatty acids, and structural lipids, phenolic compounds attract the most research interest [8]. The importance of this group of compounds is evidenced by the increasing number of reviews [9,10,11] describing nutraceuticals from structural, medical, and biotechnological aspects. Nutraceuticals also include enzymes, organic acids, vitamins, and minerals. In general, nutraceuticals play an important role as anti-aging agents, antioxidants, anticancer agents, hypoglycemic and hypocholesterolemic agents, antidepressants, etc. The spread of nutraceuticals is increasing worldwide because they are considered safe and free of side effects. The discovery of nutraceuticals is progressing inexorably, but the problem lies in the methods of their production since they are often based on the use of various environmentally harmful chemicals. In addition, there are no regulatory measures for their consumption [12].

The challenge of improving the quality of BSG for use in the food and feed industry is the fact that some components, which are potential nutraceuticals, are bound to lignin, cellulose, and/or hemicellulose or are entrapped inside of those molecules. Different treatments affect the nutritional value of foods and the bioavailability of biologically active substances. Therefore, it is desirable to choose an appropriate technique to increase or maintain the biologically active properties of certain substances [13]. Further to that, biological processing, e.g., solid-state fermentation (SSF), is one of the most important, while being considered a “green chemistry” approach, due to the reduced use of energy and chemicals, and the avoidance of the formation of toxic compounds [14]. The success of SSF is influenced by several factors depending on the nature of the substrate and the microorganism, as well as the scale-up of the process. The most commonly used microorganisms in SSF are fungi, because SSF simulates the natural conditions of the fungal habitat [15,16]. Production of enzymes by SSF at a higher level for commercial interest can be achieved with a selected type of microorganism and substrate for the corresponding enzymes in the fermentation process.

BSG was used as a substrate for the cultivation of various microorganisms for the production of both pure enzymes (laccase, xylanase, cellulase, α-amylase, β-amylase, glucanases) and enzyme cocktails, as recently reviewed [1,17,18,19]. Because of its lignocellulosic structure and availability of free sugars, it is a source of nutrients and a solid support for the growth of the selected microorganism [20]. During the fungal-based SSF process, which very often includes white-rot fungi, enzymes are synthesized and break down the structure of the substrate and release the trapped nutrients, improving the nutritional value of BSG [21]. White-rot fungi are a physiological group of basidiomycetes with an extracellular lignolytic enzymatic system, which causes wood decay in nature. Fungi and invertebrates are the dominant eukaryote taxa colonizing dead wood, in terms of both abundance and species richness, and they are the key agents of wood decomposition. However, with the exception of termites, the direct effect of invertebrates on wood decay seems to be minor relative to that of fungi [22].

*Trametes versicolor* is white-rot fungus. Based on the four-classic division (Homobasidiomycetes, Heterobasidiomycetes, Urediniomycetes, and Ustilaginomycetes), it belongs to Homobasidiomycetes [23]. According to the Index Fungorum [24], there are 892 species of *Trametes*. *Trametes versicolor* (L.) Lloyd (1921) is one of the most widespread species of *Trametes.*

*T. versicolor* is heterotrophic organism. It is a saprophyte and uses absorption as the mechanism for feeding, and forms non-motile mycelium of hyphae. When found in nature, *T. versicolor* does not have a stalk, only a cup that attaches directly to the tree or log on which it lives. It has a series of multicolored stripes across the conk. The darker stripes are covered in very small hairs which can help separate *T. versicolor* from similar fungi. Its texture is also very tough and leathery compared with many other fungi which have more delicate skins. It does not have gills, but rather pores [25].

There is a lot of research dealing with the cultivation of *T. versicolor* in different ways (solid-state and submerged fermentation) and with different purposes, as reviewed in our recent review paper [14]. In the last decade, the number of research has increased rapidly, mainly because of ecology and the possibility of reusing widely available lignocellulosic materials as substrates for *T. versicolor* growth for the purpose of producing biofuels and/or value-added products.

In this work, SSF was used for the transformation of two chemically different types of BSG originating from two different breweries. This is the continuation of our work where we have proven that *T. versicolor* produces laccase during cultivation on BSG and influence the liberation of total phenolic compounds [26]. In this work, a complex enzymatic system of *T. versicolor* hydrolytic and lignolytic enzymes was analyzed and a complete chemical analysis of the BSG was done, for each day of fermentation, supported by FTIR and NMR spectra measurements. Analysis and evaluation of the safety-related correctness of treated BSG for a possible use as a feed was done at the end.

## 2. Materials and Methods

**Scheme 1:**
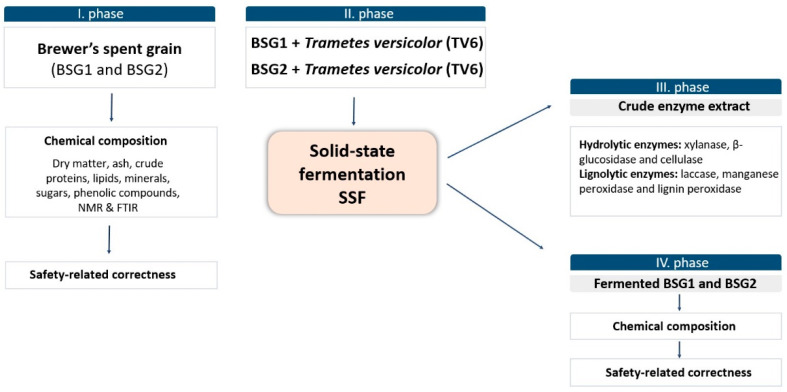
Schematic representation of the experimental setup.

The schematic overview of the experimental set-up is presented in Scheme 1.

### 2.1. Substrates and Microorganisms

Samples of BSG were kindly provided by two local breweries from Croatia, and are referred to in the paper as BSG1 and BSG2. On the day of preparation, a fresh BSG sample with a moisture content of 75% was collected and dried for 8 h at 45 °C in a ventilated oven. Until use, the dry sample was stored at 25 °C. *T. versicolor* TV-6 (MZKI, Ljubljana, Slovenia) was cultivated on potato dextrose agar (PDA) medium at 27 °C for 14 days.

### 2.2. Biological Treatment of Brewer’s Spent Grain by Trametes versicolor

SSF fermentation conditions were performed according to our previous work [26] with applied modifications. In the first step of SSF, 30 g of BSG was added to 720 mL-laboratory jars and mixed with 50 mL of distilled water. It was autoclaved at 121 °C for 15 min and then cooled. The substrate was sterilized (*T* = 121 °C/15 min), cooled, and inoculated. The inoculum contained 5 mycelial discs (Ø = 1 cm) of *T. versicolor* and 10 mL of sterile water. The height of the inoculated substrate in the laboratory jar was 4.5 cm with a moisture content of 70%. After each day of fermentation, the sample was removed from the laboratory jar and weighed to determine enzyme activity, and the fermentation residue was sterilized. The sterilized residue was dried at room temperature for 48 h and ground on an ultracentrifugal mill (Retsch ZM200, Haan, Germany) to a particle size of ≤1 mm.

### 2.3. Enzyme Activities Measurements

#### 2.3.1. Preparation of Crude Enzyme Extract

For each day of SSF, 2 g of the homogenized sample was weighed from a laboratory jar (in duplicate) and extracted in 10 mL of the appropriate buffer for each enzyme. Extraction was performed on a vortex for 30 min followed by centrifugation at 10,000× *g* (Z 326 K, Hermle Labortechnik GmbH, Wehingen, Germany). Enzyme activities were measured from the supernatant according to the tests described in Section 2.3.2.

#### 2.3.2. Measurements of Enzyme Activities

All enzyme activities were measured in triplicate for each extract using a spectrophotometer (UV-1280, Shimadzu, Kyoto, Japan). Xylanase (endo-1,4-β-xylanase) and cellulase (endoglucanases and exoglucanases) activities were determined by the DNS method [27,28], and β-glucosidase activity was determined according to the study by Karpe et al. (2016) [29]. Manganese peroxidase activity was expressed as the difference between the total and laccase activities, which were also measured under the same conditions [30]. The assay for laccase activity was done according to the study by Tišma et al. (2012) [31]. The assay method for lignin peroxidase was performed following the work by Tien et al. (1988) [32].

#### 2.3.3. pH Measurement

A total of 2 g of fermented BSG was suspended in 10 mL of distilled water. The mixture was vortexed for 30 min and pH was measured with a pH meter (HI 2211 pH/ORP Meter, Hanna instruments).

### 2.4. Chemical Composition of BSG

The chemical composition of untreated samples (BSG1 and BSG2) and treated samples for each day of fermentation was taken according to Section 2.4.1–Section 2.4.7 NMR and FTIR spectra were completed for untreated samples (BSG1 and BSG2) and treated samples BSG1 (day 10) and BSG2 (day 15).

#### 2.4.1. Dry Matter Content

The percentage of dry matter was determined with a rapid moisture analyzer (HR-73, Mettler Toledo, Zürich, Switzerland) according to the thermogravimetric method [33].

#### 2.4.2. Ash Content

The ash content was analyzed by the gravimetric method AACC-08-03 [34].

#### 2.4.3. Crude Proteins

Protein was determined by the Kjeldahl method using the Kjeldahl digestion unit (Behr Labor-Tecnik, Behrotest K12, Düsseldorf, Germany) and water steam distillation system (Gerhard, Vapodest 1, Königswinter, Germany) [35].

#### 2.4.4. Lipids

Lipid content was determined according to the Soxhlet method [35] using an extraction principle (Büchi B-811 LSV, Flawil, Switzerland).

#### 2.4.5. Neutral Detergent Fibers (NDF), Acid Detergent Fibers (ADF), and Acid Detergent Lignin (ADL)

NDF, ADF, and ADL were determined according to the Van Soest method [36] using a fiber analyzer (FIWE 3, VELP Scientifica, Usmate Velate, Italy). The dried and ground sample was weighed (1.0000 ± 0.0001 g) and placed in glass crucible that had previously been dried at 105 °C for 1 h and cooled in a desiccator. For the determination of NDF, the samples were refluxed for 60 min, adding 100 mL of NDF solution, 0.5 g of Na_2_SO_3_, and a few drops of n-octanol. Samples were then filtered with boiling water and acetone, dried at 105 °C for 8 h, cooled, and weighed. For ADF, 100 mL of ADF solution and a few drops of n-octanol were added and the procedure was repeated as for NDF. To perform ADL analysis, the ADF analysis was performed first and 25 mL of 72% H_2_SO_4_ was added to the glass wells containing the filtered samples and washed with boiling water and cold acetone. Cold extraction was then performed for 3 h, stirring the samples every hour. At the end of the extraction, the samples were washed with boiling water until the acid was no longer present. The samples were also dried at 105 °C for 8 h, cooled in a desiccator, and weighed. NDF, ADF, and ADL were calculated and corrected for ash by burning glass crucibles with samples in a muffle furnace at 550 °C for 2 h, cooling, and weighing. Hemicellulose content was calculated as the difference between NDF and ADF, cellulose content as the difference between ADF and ADL, and ADL represents lignin content.

#### 2.4.6. Content of Total Reducing Sugars

Total reducing sugars were measured spectrophotometrically (UV-1280, Shimadzu, Japan) at 540 nm according to the DNS method [37].

#### 2.4.7. Content of Total Phenolic Compounds, Total Flavonoids and Total Extractable Proanthocyanidins and Individual Phenolic Compounds

The determination of total phenolic compounds was performed spectrophotometrically using the Folin–Ciocalteu method [38]. Total flavonoids were also determined spectrophotometrically by the aluminum chloride method [39]. Total extractable proanthocyanidins were determined using an acid-butanol solution by a modified spectrophotometric method [40]. All analyses were carried out in duplicate. Analysis of individual phenolic compounds in BSG was determined by ultra-high liquid performance chromatography according to Šelo et al. (2022) [33].

#### 2.4.8. FTIR and NMR Spectra

The FTIR spectra of BSG samples were recorded on a Shimadzu Tracer 100 spectrometer using the ATR (attenuated total reflectance) technique in the single reflection configuration between 4000–400 cm^−1^ with a resolution of 4 cm^−1^ and 128 scans. Solution state 1 H NMR spectra were performed on a Bruker Avance 300 NMR spectrometer using a C/H dual 5 mm probe in D_2_O (99.8%, TCI) at room temperature. 1H NMR spectra were recorded with 10 s, 7.6 μs π/2 pulse length, 16 K time domain, and 100 scans.

Solid-state NMR spectra of BSG samples were measured on a Bruker Avance NEO 400 spectrometer using a broad-band magic angle spinning (MAS) probe. The samples were spun in a 4 mm rotor with a 10 kHz spinning rate. 1H MAS spectra were recorded with 10 scans, 3 s relaxation delay, 0.40 s acquisition time, and 2.5 μs excitation pulse length. 13C cross-polarization (CP) MAS NMR experiments were performed with a standard CP MAS pulse sequence and high-power decoupling during acquisition. The spectra were recorded with 10,000 scans, 3 s relaxation delay, 27 ms acquisition time, and 1 ms contact time. During the contact time, variable amplitude CP ramped from 70% to a maximum of 80 kHz. The protons were decoupled using the SPINAL-64 decoupling. Both 1H MAS and 13C CP MAS spectra were externally referenced to glycine.

#### 2.4.9. Analysis and Evaluation of the Safety-Related Correctness of BSG for Feed

All analyses were performed according to the appropriate accredited method. Detection of nitrates and nitrites was performed by HPLC according to the RU-308-01 method, and gross energy was determined calorimetrically according to HRN EN ISO 9831:2004. Phosphorus content was analyzed spectrophotometrically according to HRN ISO 6491:2001 method, calcium, potassium, iron, lead, cadmium, and arsenic according to the HRN EN ISO/IEC 17025:2017, RU-305-05 on ICP-MS. The analysis for undesirable substances such as mycotoxins was carried out according to the RU-287-04 method (LC-MS/MS), ergot alkaloids RU-149-04 (LC-MS/MS), tropane alkaloids RU-418-01 (LC-MS/MS), polychlorinated biphenyls RU-231-04 (GC-MS/MS), and polycyclic aromatic hydrocarbons RU-230-03; RU-256-02 (GC-MS/MS).

## 3. Results and Discussion

### 3.1. Cultivation of Trametes versicolor on Brewer’s Spent Grain

In this work, the cultivation of *T. versicolor* on brewer’s spent grain in SSF was performed for 15 days in laboratory jars. Moisture content is extremely important in the SSF process because the solid substrate must have adequate available moisture to allow microbial growth and metabolism. Water content that is too high would result in the compaction of the solid, which would introduce the possibility of contamination and prevent oxygen transport. On the other hand, low water content limits nutrient transport and enzyme production [41]. The moisture content of BSG1 during the 15-day fermentation ranged from 66.89% at the beginning to 67.49% at the end of fermentation, and for BSG2 from 69.97% to 67.49%, which is the optimal humidity required for *T. versicolor* growth as was already proved in our previous works. The initial moisture content of BSG was 63% [26] while those of corn silage was 75% [42] and grape pomace was 65–75% [33]. In a study by Iqbal et al. (2011) [43] rice straw used as a substrate for *T. versicolor* had 66.6% of moisture content.

There was no need to add an extra amount of water during SSF while the rate of water production during fungal metabolism was slightly faster than the rate of water evaporation in the case of BSG1. With BSG2, the opposite situation occurred, where water evaporation was slightly faster which lead to a decrease in moisture content, but not below the undesired level.

As a result of organic matter degradation, the loss of the total substrate mass after 15 days of fermentation was 28.74% for BSG1 and 31.01% for BSG2.

The pH analysis showed an increase in the value of fermented BSG1 from an initial 5.44 to 6.11 at the end of fermentation. Fermented BSG2 showed an insignificant decrease from the initial 5.91 to 5.75.

### 3.2. Trametes versicolor Enzymatic Activities during Cultivation on Brewer’s Spent Grain

During SSF cultivation, *T. versicolor* produces a variety of enzymes involved in lignocellulose degradation and modification. In this work, the enzymatic activities of hydrolytic enzymes (xylanase, β-glucosidase and cellulase) and lignolytic enzymes (laccase, manganese peroxidase and lignin peroxidase) were measured during 15 days of SSF.

Aside from being involved in the degradation and modification of lignocellulosic materials, some of those enzymes (e.g., laccase) belong to industrially important enzymes. SSF is gaining more and more attention for the possibility to be used for the production of industrially important enzymes, due to a number of advantages, compared to submerged fermentation. Some of them include the simplicity of implementation, lower production costs, high enzyme productivity, and a positive impact on the environment. However, in addition to the advantages, the SSF also encounters obstacles such as a difficult scale-up, problems in ensuring sterility, heterogeneity of the reaction mixture, oxygen transfer limitation and heat accumulation, variability of conditions during the process, and the effect of inducers [14,44].

**Figure 1:**
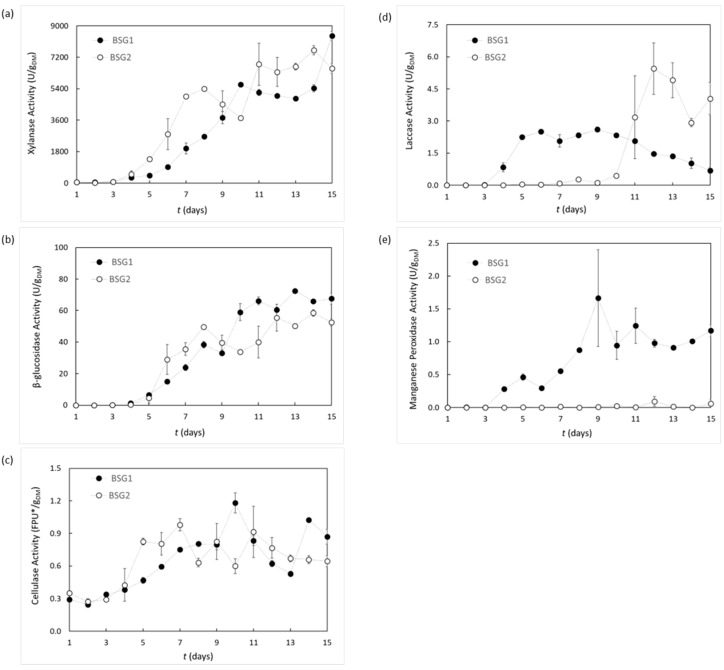
Time course of enzyme activity (**a**) xylanase, (**b**) β-glucosidase, (**c**) cellulase, (**d**) laccase, and (**e**) manganese peroxidase produced by *T. versicolor* on BSG. Activities are expressed as U per gram of dry BSG or * FPU (filter paper unit) per gram of dry BSG.

The results of enzymatic activities during the cultivation of *T. versicolor* on BSG are shown in Figure 1. Concentrations are expressed as mean value ± standard deviation.

The maximum xylanase productivity of 8430.43 U/g_DM_ was reached on day 15 for BSG1, while for BSG2, the maximum activity of 7608.74 U/g_DM_ was reached on day 14. These results are higher if compared with other microorganisms cultivated on BSG. For example, during cultivation of *Penicillium janczewskii* on BSG, a maximum xylanase activity of 371 U/g_DM_ was achieved on day 7 [45], while cultivation of *Aspergillus niger* strains lead to a maximum xylanase activity of 1400.80 U/g_DM_ [46]. According to the literature, the optimized conditions (pH 6, *T* = 30 °C, *m*(BSG) = 15 g) for xylanase production from BSG with *Mucor* species resulted in significantly lower activity values of 67 U/gDM, compared to our results [47]. However, other hydrolytic enzymes (β-glucosidase and cellulase) were less active. The maximum activity of β-glucosidase for BSG1 was 72.55 U/g_DM_ on day 12 of SSF and for BSG2 was 58.56 U/g_DM_ on day 13 of SSF. The maximum cellulase activity of 1.18 U/g_DM_ was reached for BSG1 after 11 days of SSF, and for BSG2 it was 0.92 U/g_DM_, after 13 days. Similar results were obtained with *Aspergillus brasiliensis* when cultivated on BSG. The obtained β-glucosidase activity was 19.02 U/g_DM_ BSG using 1 × 10^6^ spores or 6.64 U/g_DM_ BSG using 25 × 10^6^ spores) while activities of cellulases were 7.26 U/g_DM_ BSG using 1 × 10^6^ spores or 2.92 U/g_DM_ BSG using 25 × 10^6^ spores [48].

The combination of *T. versicolor* and BSG under SSF conditions resulted in the successful production of laccase and manganese peroxidase, but proved unsuccessful for lignin peroxidase, while its activity was below the detection limit throughout the whole fermentation process. The maximum activity of laccase (BSG1) was reached after 9 days of fermentation with a value of 2.61 U/g_DM_ and for BSG2 with 5.45 U/g_DM_ after 12 days of fermentation. It was shown that the laccase activities on BSG, produced by *Bjerkandera adusta* and *Schizophyllum commune* reached 1.44 U/g_DM_ and 2.79 U/g_DM_, respectively [49]. Relatively low activities for manganese peroxidase were obtained in both experiments, with a maximum activity of 1.66 U/g_DM_ after 9 days for BSG1 and for BSG2 of 0.09 U/g_DM_ after 12 days of fermentation.

### 3.3. Chemical Composition of Brewer’s Spent Grain

Since the chemical composition depends on the process of obtaining BSG, i.e., the type of beer and the brewing process, the type of malt used in beer production, the type of barley, and the time of harvest [50], there are variations in the composition of different BSG.According to the literature, BSG is comprised of 15.2–28.7%_DM_ of cellulose, 19–20%_DM_ of hemicellulose, and 3.35–11.41%_DM_ of lignin. It also consists of crude proteins with an average value of 21.25%_DM_, lipids of 8.4%_DM_ and ash of 3.7%_DM_ [1].

#### 3.3.1. Chemical Analysis of the Fibers, Proteins, Lipids and Ash Content

Table 1 shows the chemical composition (cellulose, hemicellulose, lignin, crude proteins, lipids, and ash) of raw samples of BSG1 and BSG2 and fermented BSG1 and BSG2 after 15 days of SSF.

The analysis of the fiber content results shows that the lignin, cellulose, and hemicellulose content decreased after SSF in BSG1, whereas in BSG2 cellulose content decreased, while the hemicellulose and lignin content increased. The reason for this is that BSG2 contains a higher amount of simple sugars (Section 3.3.2), which *T. versicolor* uses for its growth before other carbon sources. It is assumed that, in this case, a longer incubation period is required for the fungus to begin consuming more complex carbohydrates as a source of growth, with possible degradation. Although xylanase activities were high in both experiments, hemicellulose content decreased only in the BSG1 experiment and increased in the BSG2 experiment. It is likely that other types of enzymes besides those investigated in this study are involved in the modification of BSG. Generally, the use of processed BSG with reduced fiber content in animal feed may improve digestibility in animals that cannot digest fiber [48]. THe protein content increased 1.15-fold for BSG1 and 1.38-fold for BSG2 after 15 days of fermentation, which contributes to the quality of BSG in the form of protein-enriched animal feed. The results of the work by Eliopoulos et al. (2022) [4] demonstrate that SSF has a positive effect on improving protein content of BSG, where the protein content reached 25.01% after 12 days of SSF with *Pleurotus ostreatus*, compared with 16.73% in untreated BSG. SSF with the fungus *Rhizopus* species leads to an increase in protein content from the initial 20.5% to 31.7% after 9 days of SSF [51]. A reduction in lipid content was observed in both BSG1 and BSG2 after 15 days of SSF. Similar results were obtained in the fermentation of BSG with *Rhizopus oligosporus*, where the lipid content decreased from the initial 10.9% to 4.52% after 3 days of SSF [21]. SSF resulted in a 1.30-fold increase in ash content in BSG1 and a 1.35-fold increase in BSG2, with the increase in minerals occurring during organic matter degradation by fermentation. The results of mineral composition are presented in Section 3.3.5.

The results of this study are consistent with the comprehensive literature review [17], which presented that the conversion of BSG involving natural lignocellulosic decomposers by the SSF process significantly improves the nutritional composition of BSG by increasing the content of amino acids, vitamins, and antioxidants while decreasing the content of carbohydrates, fat, and fiber.

#### 3.3.2. Total Reducing Sugars

Various white rot fungi, including *T. versicolor*, are reported in the literature to be good players in removing lignin and hemicellulose with a somewhat lesser effect on cellulose, in lignocellulosic materials. The action of these fungi results in the release of sugars from the mentioned components, e.g., a sugar conversion of 13% was achieved in wheat straw by *P. ostreatus*, *Phanerochaete sordida*, and *Pycnoporus cinnabarin* [52].

**Figure 2:**
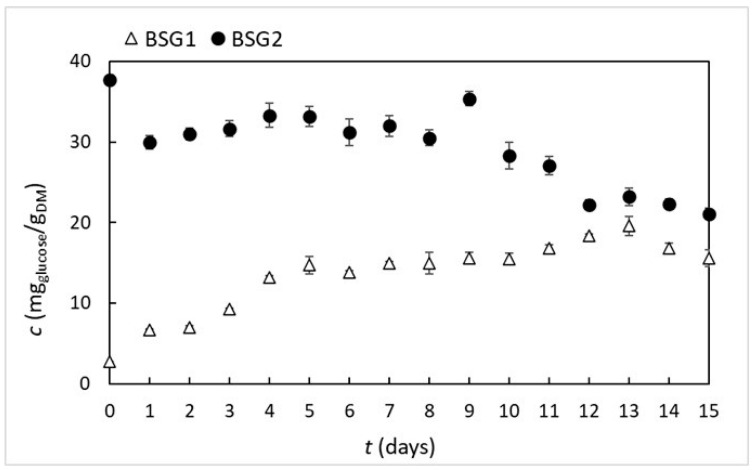
Time course of total reducing sugar concentration during SSF with BSG by *T. versicolor*.

The initial concentration of total reducing sugar varies significantly between BSG1 and BSG2 in this work, and the results are shown in Figure 2. Concentrations are expressed as mean value ± standard deviation.

The concentration of total reducing sugar of BSG1 was 2.72 mg/g_DM_, whereas in BSG2 it was 37.66 mg/g_DM_. This wide range and difference between BSG1 and BSG2 can possibly be attributed to the type of raw materials used in the process and the applied brewing process. Variations in total reducing sugar content in BSG samples have also been reported in the literature. For example, Llimós et al. (2020) [53] measured 30 mg/g_DM_ in a raw BSG sample, and Fernandes et al. (2021) [20] determined 6.7 mg/g_DM_.

Regarding the concentration of total reducing sugars, SSF fermentation resulted in an increase in the concentration of reducing sugars in BSG1 after 13 days of fermentation with a maximum concentration of 19.56 mg/g_DM_, probably due to the action of the enzymes produced and the degradation of other polysaccharide components. In BSG2, the concentration of total reducing sugars decreases to 21.07 mg/g_DM_ at the end of fermentation. In this case, it can be assumed that the fungus prefers to consume fermentable sugars and uses them predominantly as an energy source for its growth [4].

#### 3.3.3. Total and Individual Phenolic Compounds, Total Flavonoids, and Total Extractable Proanthocyanidins

In general, the composition of polyphenolic compounds in raw BSG depends on the production process, especially the brewing temperature and the type of malt used (light and dark) [50]. Due to the large number of enzymes produced during the SSF process, compounds bound to the cell wall are released in the soluble extract, possibly altering the phenolic profile in BSG [54].

The results of the measurement of total polyphenols, flavonoids, and proanthocyanidins are shown in Table 2. for raw and fermented BSG. The individual polyphenolic compounds analyzed by UHPLC are also listed.

The rather low total polyphenol contents in raw BSG are related to the fact that barley does not originally contain a large number of polyphenols and these compounds are associated with lignin and cell wall polysaccharides, so it is necessary to degrade lignocellulosic structures to release polyphenolic compounds [55]. To this purpose, SSF with *T. versicolor* proved successful in the release of total and individual polyphenolic compounds. Total polyphenolic compounds increased 3.75-fold in BSG1 and 1.64-fold in BSG2 by fermentation. Total flavonoids also increased 1.62-fold in BSG1 and 1.14-fold in BSG2. SSF had no positive effect on the concentration of total extractable proanthocyanidins. The increase in total polyphenolic compounds may be attributed to β-glucosidases produced during bioprocessing by the *T. versicolor*. These enzymes hydrolyze β-glucoside bonds to allow free phenolic compounds to react with the Folin–Ciocalteau reagent [15]. In our previous work on BSG with *T. versicolor*, a four-fold increase in total polyphenolic compounds was achieved after 14 days of fermentation, from an initial 2.5 mg/g_DM_ to the final value of 8.7 mg/g_DM_ [26].

The phenolic compounds more commonly found in BSG are hydroxycinnamic acids, especially ferulic acid and *p*-coumaric acid, sinapic acid, caffeic acid, and syringic acid [56]. Our collection includes a total of 27 standards for polyphenolic compounds, 11 of which were detected in this study. For all detected individual polyphenolic compounds, an increase was observed at a particular stage of SSF, with 3,4-dihydroxybenzoic acid in BSG1 standing out the most with a 14.77-fold increase in concentration.

#### 3.3.4. FTIR and NMR

**Figure 3:**
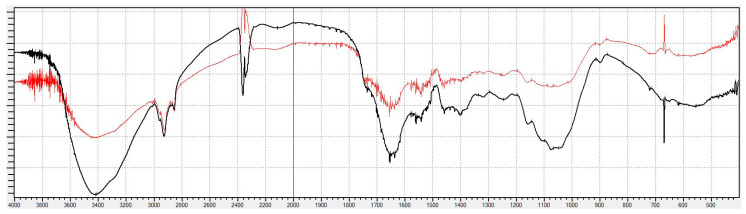
The typical FTIR spectra of raw (red) and fermented (black) BSG samples.

The typical FTIR spectra of raw (black) and fermented (red) are displayed in Figure 3.

**Figure 4:**
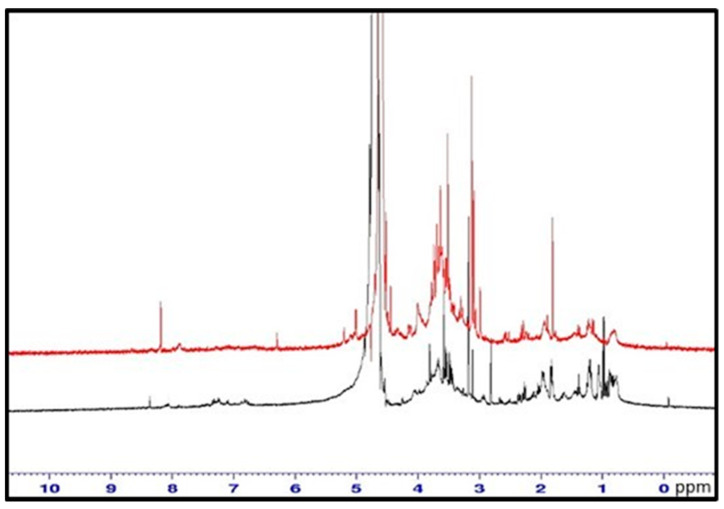
The typical ^1^H NMR spectra of raw (black) and fermented (red) BSG samples.

FTIR spectroscopy was used to assess the composition of BSG samples. All samples showed broad adsorption bands in the region between 3000 and 3800 cm^−1^, belonging to the stretching vibrations of the phenolic and aliphatic –OH groups and between 2800 and 3000 cm^−1^, attributed to C–H stretching of methyl and methylene groups Furthermore, –C=O (carboxylic acids, and esters) and –C=C stretching bands were observed in the region 1600–1750 cm^−1^, while C–O–C and C–O vibrations were found in the region between 1000 and 1250 cm^−1^. C–H deformation, aromatic ring vibration, and –OH bending vibration bands span the region between 1000 and 1500 cm^−1^. Proton NMR spectra in a solution of raw and fermented BSG samples are presented in Figure 4. The characteristic spectral regions and corresponding functional groups (for all analyzed samples) have been assigned and compared with the data already published in the literature (Table 3) [57,58,59,60,61].

Despite the fact that BSG components were not separated and the complexity of proton NMR spectra, some significant differences in composition among samples can be noticed. According to the integrated proton peak intensities in NMR spectra, fermented BSG samples seem to show a higher content of aliphatic and aromatic compounds and a lower portion of carbohydrates, amino, and formic acids compared to raw BSG samples.

BSG samples were further analyzed by solid-state NMR using ^1^H and ^13^C CP MAS techniques. Carbon and hydrogen atoms were assigned based on already published NMR data and comparison with solution state spectra [58,59,60].

**Figure 5:**
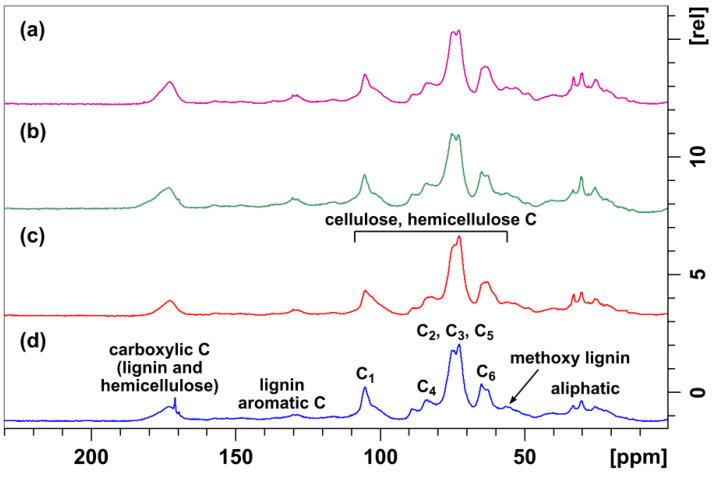
^13^C CP MAS spectra of (a) raw BSG1; (b) fermented BSG1; (c) raw BSG2; (d) fermented BSG2.

As can be seen in Figure 5, the most intense signals in the spectra were found in the region between 60 and 110 ppm. This region belongs to the carbohydrates of cellulose and hemicellulose. Aromatic and aliphatic signals are associated with carbon atoms of lignin. The broad signals observed approximately at 55 ppm were attributed to methoxy carbon atoms of lignin. The most deshielded peaks, observed between 169 to 175 ppm, were assigned to carboxylic carbon atoms of lignin and hemicellulose.

**Figure 6:**
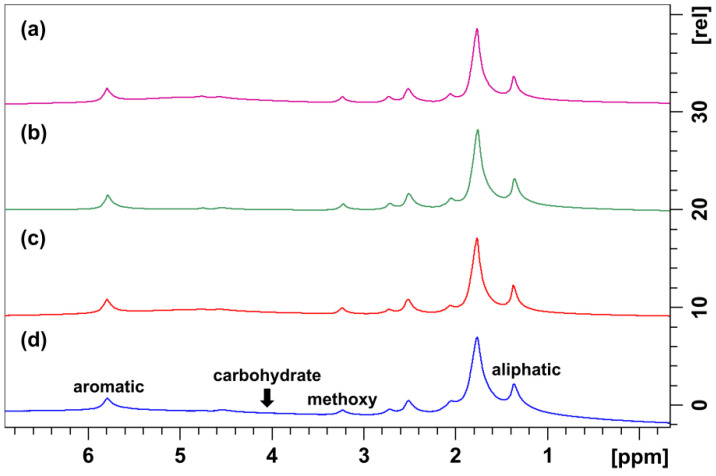
^1^H MAS spectra of (a) raw BSG1; (b) fermented BSG1; (c) raw BSG2; (d) fermented BSG2.

The ^1^H chemical shift assignment in the solid state was made on the basis of the comparison with chemical shifts observed in the solution (Figure 6).

#### 3.3.5. Analysis and Evaluation of the Safety-Related Correctness of BSG for Feed

When BSG is considered a potential addition to animal feed, functional food for humans, or plant food in the form of biofertilizer, it is necessary to examine the parameters that affect the quality of correctness [17]. Research shows that BSG may contain significant amounts of mycotoxins, secondary metabolites originally found in grains that can also be produced by fungi, particularly *Aspergillus*, *Fusarium*, and *Penicillium*. They cause food spoilage and are of significant concern to humans and animals [62,63]. The consumption of feeds containing mycotoxins causes adverse health effects in animals (stunted growth, reduced immunity, chronic and acute diseases, and death) and leads to restrictions in livestock production [64].

The total content of aflatoxins in raw and fermented BSG is <0.003 mg/kg, fumonisins < 0.30 mg/kg, zearalenone < 0.030 mg/kg, T-2 and HT-2 sum < 0.20 µg/kg, and ochratoxin A < 0.001 mg/kg expressed as Limit of Quantification (LOQ).

Other secondary metabolites found in commercially important cereals are ergot alkaloids produced by fungi of the genus *Claviceps* that cause ergot disease. Studies show that ergot alkaloids are often present in animal feed at mean concentrations ranging from 25–96 µg/kg to a maximum of 149–4883 µg/kg [65]. In this work, 12 ergot alkaloids were analyzed with a total concentration of less than 120 µg/kg (LOQ) in all BSG. Cereals may also be contaminated with solanaceous plants (*Solanaceae*), which contain toxic metabolites such as tropane alkaloids. The LOQ for these compounds in feed was 5–25 μg/kg in the study [66], while in this study, atropine and scolopamine were analyzed and their concentration was <1.0 μg/kg LOQ in raw and fermented BSG.

Polychlorinated biphenyls (PCBs) in the feed are a group of complex substances that can affect the hormonal, nervous, and immune systems of animals. Studies show that PCB concentrations in feed were below the LOQ (0.05 mg/kg) in 30 feed samples [67]. The total PCB LOQs in this work were <0.005 mg/kg for raw BSG1 and <0.035 mg/kg for fermented BSG1. For raw and fermented BSG2, the concentration was <0.035 mg/kg. Other hazardous compounds that have carcinogenic and mutagenic properties are polycyclic aromatic hydrocarbons (PAHs), the contaminants from the air, water, and soil. Animals exposed to PAHs experience disorders of the immune system, urinary system, body fluids, and skin and lung damage. In studies of feed mixtures for pigs and cows, PAHs were detected at levels of 0.082 and 0.128 mg/kg, respectively [68], while in this study they were < 0.020 mg/kg LOQ for raw and fermented BSG.

The formulation of animal feed requires a certain amount of minerals necessary to maintain animal health and productivity. Many essential trace elements (Fe, I, Co, Zn, Cu, Mn, Mo, Se) are added to animal feed as dietary supplements, while some (As, Cd, F, Pb, Hg) are considered undesirable substances and have no proven biological functions [69]. The elements analyzed in raw and fermented BSG1 and BSG2, as well as gross energy, are listed in Table 4. For BSG1, the selected analyzes were performed on the sample on the 10th day of fermentation, while for BSG2 the 15th day was selected. The reason for selecting these days was the sum of all the results and enzyme productivity, and the days with the most of the best results were selected.

From the results, fermentation releases minerals in the BSG material as the ash content increases, which makes this material attractive for addition to animal feed due to mineral enrichment of the feed composition.

According to the EU DIRECTIVE 2002/32/EC [70], the maximum permissible level of arsenic in feed materials and complementary feed is 2 mg/kg, for lead 10 mg/kg and for nitrites 15 mg/kg. The permissible concentrations of cadmium in feed materials of vegetable origin and complementary feed are 1 and 0.5 mg/kg, respectively. Table 4 shows that the heavy metals in raw as well as in fermented BSG1 and BSG2 are within the permissible concentration limits.

## 4. Conclusions

The use of *T. versicolor* with BSG under SSF fermentation conditions resulted in the successful production of hydrolytic and lignolytic enzymes, with particular emphasis on xylanase, for which the highest activities were obtained. The production and action of enzymes during the fermentation process resulted in an increase in protein content, ash content, polyphenolic compounds, and sugars in BSG1, while in BSG2 the overall reducing sugar content decreased. The degradation of cellulose, hemicellulose, and lignin as a result of SSF is observed in BSG1, while in BSG2 only cellulose was degraded and the content of hemicellulose and lignin increased. Fat content also decreased in both BSGs during fermentation. These results make SSF a promising alternative for the valorization of BSG as a valuable ingredient for possible use in animal feed. Undesirable substances in animal feed have also been shown to be within acceptable limits for unprocessed and fermented BSG.

## Figures and Tables

**Table 1:** Composition of raw and fermented BSG by *T. versicolor* under SSF conditions.

Component [%]	Raw BSG1	Fermented BSG1	Raw BSG2	Fermented BSG2
Cellulose	21.47 ± 1.22	17.82 ± 1.57	28.34 ± 0.46	26.59 ± 0.75
Hemicellulose	30.95 ± 1.55	27.87 ± 0.87	14.89 ± 1.25	20.26 ± 2.39
Lignin	6.94 ± 0.95	5.75 ± 0.34	3.81 ± 0.26	6.90 ± 1.97
Crude Proteins	23.07 ± 0.13	26.42 ± 0.11	18.34 ± 0.01	25.29 ± 0.24
Lipids	8.09 ± 0.17	3.82 ± 0.19	8.85 ± 0.62	3.99 ± 0.55
Ash	3.86 ± 0.03	5.00 ± 0.00	4.24 ± 0.06	5.73 ± 0.11

**Table 2:** Concentrations of total phenolic compounds, total flavonoids, total extractable proanthocyanidins, and individual phenolic compounds in raw BSG and fermented BSG with *T. versicolor*.

*c*_max_ [mg/g_DM_] *	Raw BSG1	Fermented BSG1	SSF Day	Raw BSG2	Fermented BSG2	SSF Day
Total Polyphenols	4.495 ± 0.270	16.867 ± 0.126	14	9.242 ± 0.068	15.198 ± 0.384	7
Total Flavonoids	1.268 ± 0.025	2.056 ± 0.029	11	1.971 ± 0.044	2.246 ± 0.139	7
Total Proanthocyanidins	0.517 ± 0.431	0.287 ± 0.030	15	0.494 ± 0.009	0.456 ± 0.009	15
Individual Phenols						
*p*-hydroxybenzoic acid	0.022 ± 0.001	0.105 ± 0.003	3	0.203 ± 0.047	0.324 ± 0.008	6
Catechin	0.148 ± 0.009	1.301 ± 0.025	15	0.188 ± 0.011	1.152 ± 0.047	7
Epicatechin	0.092 ± 0.005	0.137 ± 0.010	11	0.035 ± 0.000	0.149 ± 0.001	3
Vanillic acid	0.002 ± 0.00	0.013 ± 0.000	15	n.d	n.d.	-
Syringic acid	0.031 ± 0.001	0.281 ± 0.000	9	0.015 ± 0.000	0.218 ± 0.000	7
*p*-coumaric acid	0.008 ± 0.000	0.019 ± 0.000	12	0.004 ± 0.000	0.024 ± 0.001	5
Ferulic acid	n.d.	0.139 ± 0.000	12	0.166 ± 0.027	0.164 ± 0.006	5
Kaempferol	0.023 ± 0.000	0.0230 ± 0.003	10	0.016 ± 0.000	0.040 ± 0.002	7
3,4-dihydroxybenzoic acid	0.462 ± 0.001	6.823 ± 2.159	3	0.025 ± 0.000	0.141 ± 0.008	12
*o*-coumaric acid	0.012 ± 0.001	0.019 ± 0.001	8	0.012 ± 0.001	0.019 ± 0.001	8
*p*-hydroxyphenylacetic acid	n.d.	n.d.	-	0.203 ± 0.047	0.324 ± 0.008	6

* concentrations are expressed as mean value ± standard deviation.

**Table 3:** NMR assignments and percentages of analyzed BSG samples.

δ/ppm	H-Atom	Raw BSG1/% *	Fermented BSG1/% *	Raw BSG2/%	Fermented BSG2/%
0.50–3.00	Aliphatic	29.17	31.33	25.71	30.82
3.00–4.40	Carbohydrate, methoxy	69.45	66.77	71.42	62.89
6.70–7.65	Aromatic	0.70	1.20	2.14	5.66
7.94–8.50	Aromatic, amino acid, formic acid	0.70	0.70	0.73	0.63

* calculated from integrated proton peak intensities.

**Table 4:** Minerals and heavy metals detected in raw BSG and fermented BSG with *T. versicolor*.

Compound	Raw BSG1	Fermented BSG1	Raw BSG2	Fermented BSG2
Phosphorus [%]	0.48	0.60	0.66	0.80
Calcium [mg/kg]	2073	2456	2763	2994
Potassium [mg/kg]	586	499	722	936
Iron [mg/kg]	173	220	103	113
Lead [mg/kg]	0.049	0.064	0.025	0.033
Cadmium [mg/kg]	0.039	0.048	0.044	0.057
Arsenic [mg/kg]	0.032	0.039	<0.010	0.013
